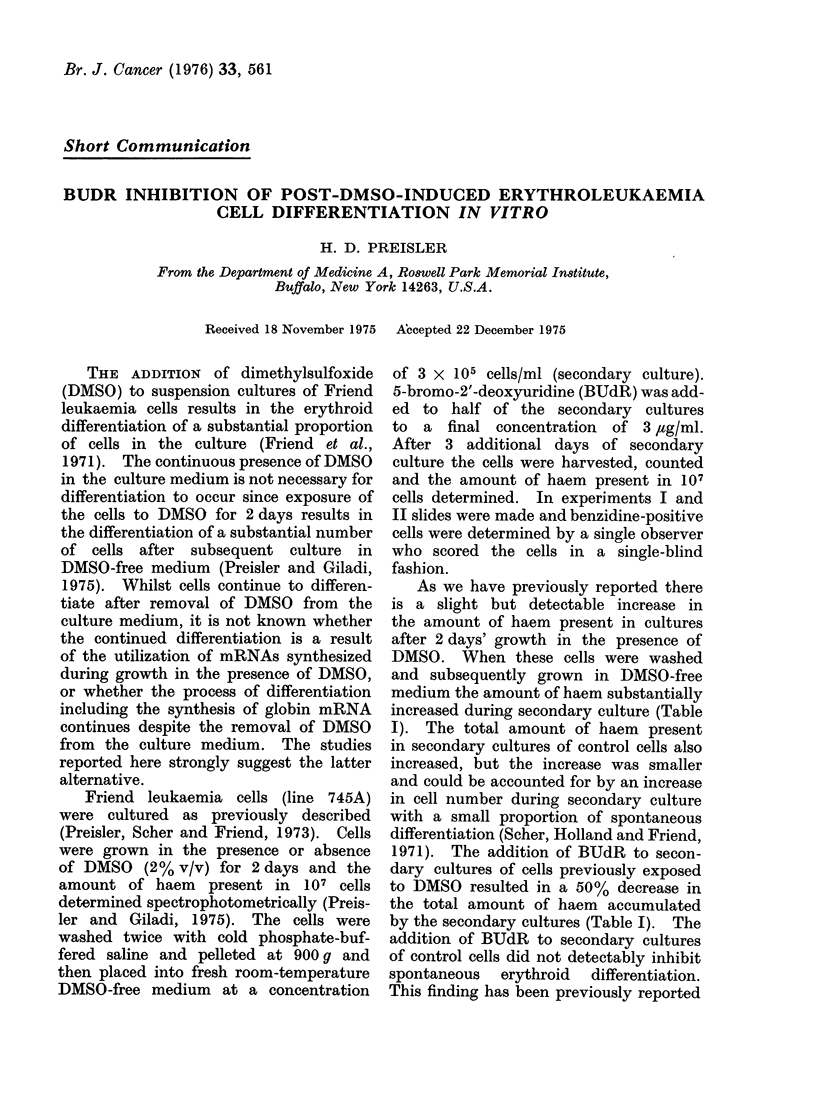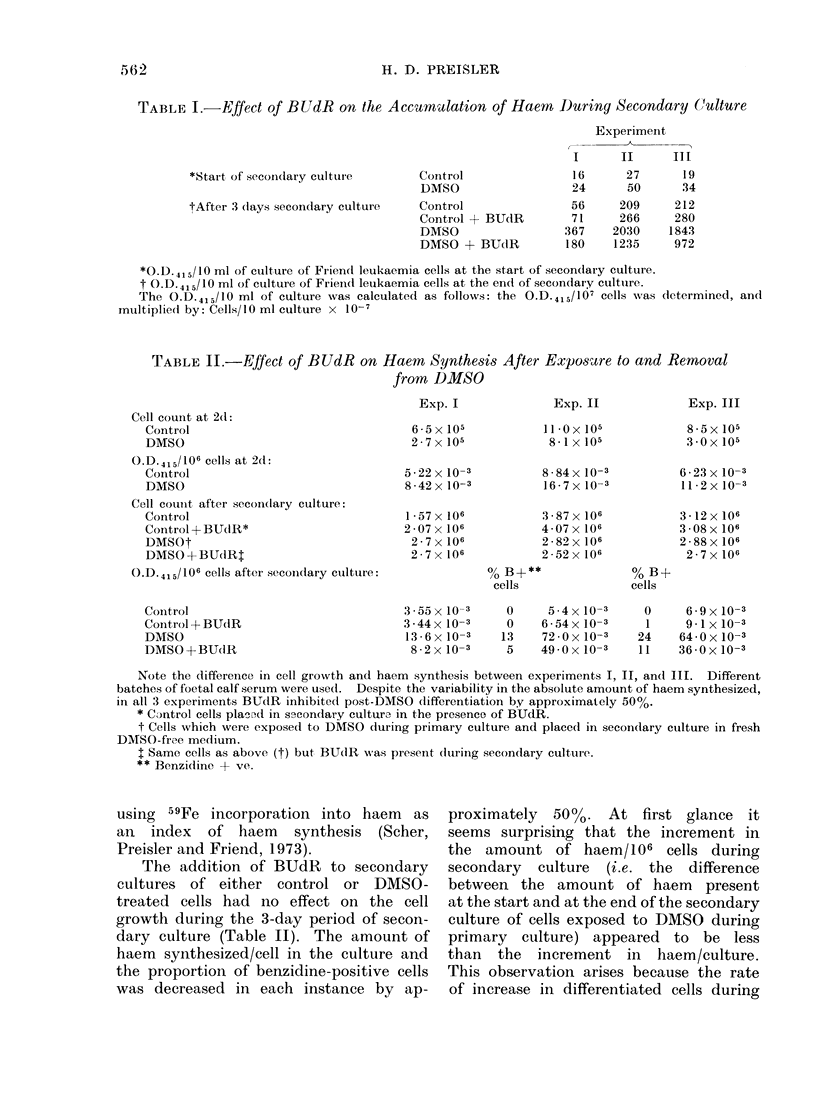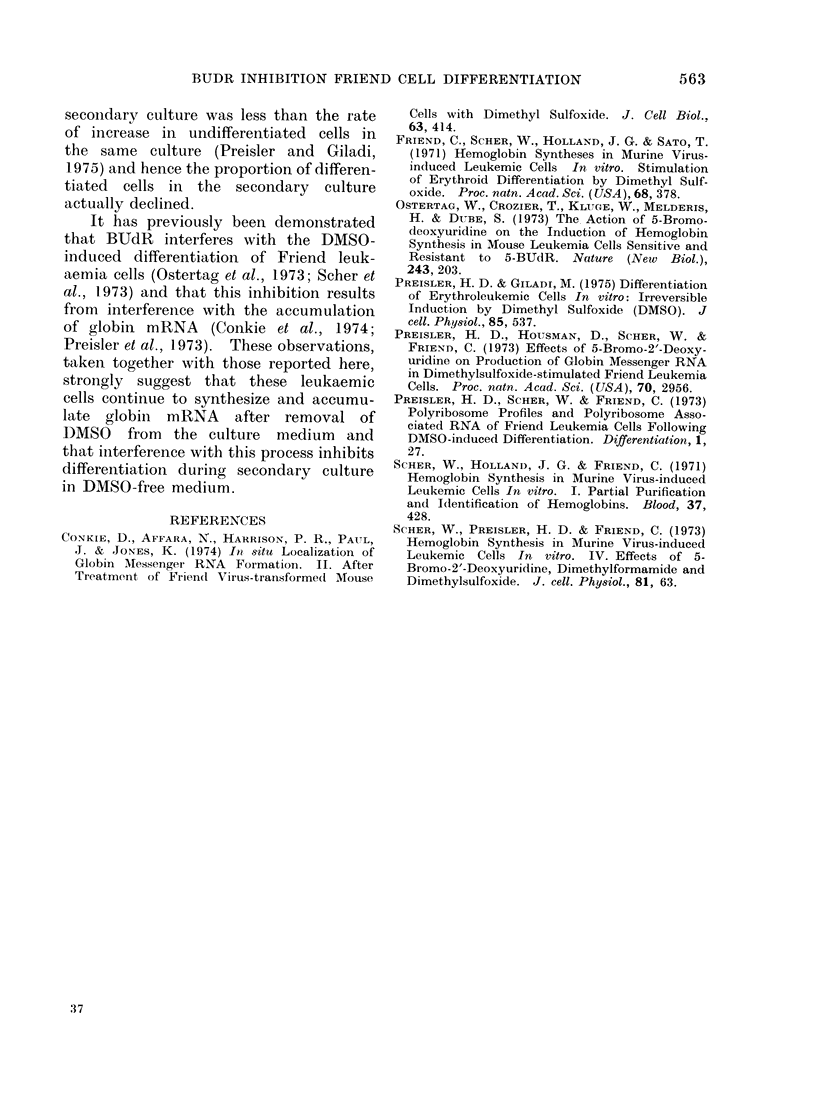# BUDR inhibition of post-DMSO-induced erythroleukaemia cell differentiation in vitro.

**DOI:** 10.1038/bjc.1976.89

**Published:** 1976-05

**Authors:** H. D. Preisler


Br. J. Cancer (1976) 33, 561

*Short Communication*

### BUDR INHIBITION OF POST-DMSO-INDUCED ERYTHROLEUKAEMIA CELL DIFFERENTIATION *IN VITRO*

H. D. PREISLER

*From the Department of Medicine A, Roswell Park Memorial Institute, Buffalo, New York* 14263, *U.S.A.*

Received 18 November 1975 Accepted 22 December 1975

THE ADDITION of dimethylsulfoxide (DMSO) to suspension cultures of Friend leukaemia cells results in the erythroid differentiation of a substantial proportion of cells in the culture (Friend *et al.*, 1971). The continuous presence of DMSO in the culture medium is not necessary for differentiation to occur since exposure of the cells to DMSO for 2 days results in the differentiation of a substantial number of cells after subsequent culture in DMSO-free medium (Preisler and Giladi, 1975). Whilst cells continue to differentiate after removal of DMSO from the culture medium, it is not known whether the continued differentiation is a result of the utilization of mRNAs synthesized during growth in the presence of DMSO, or whether the process of differentiation including the synthesis of globin mRNA continues despite the removal of DMSO from the culture medium. The studies reported here strongly suggest the latter alternative.

Friend leukaemia cells (line 745A) were cultured as previously described (Preisler, Scher and Friend, 1973). Cells were grown in the presence or absence of DMSO (2% v/v) for 2 days and the amount of haem present in $10^7$ cells determined spectrophotometrically (Preisler and Giladi, 1975). The cells were washed twice with cold phosphate-buffered saline and pelleted at 900 *g* and then placed into fresh room-temperature DMSO-free medium at a concentration of $3 \times 10^5$ cells/ml (secondary culture). 5-bromo-2′-deoxyuridine (BUdR) was added to half of the secondary cultures to a final concentration of 3 μg/ml. After 3 additional days of secondary culture the cells were harvested, counted and the amount of haem present in $10^7$ cells determined. In experiments I and II slides were made and benzidine-positive cells were determined by a single observer who scored the cells in a single-blind fashion.

As we have previously reported there is a slight but detectable increase in the amount of haem present in cultures after 2 days' growth in the presence of DMSO. When these cells were washed and subsequently grown in DMSO-free medium the amount of haem substantially increased during secondary culture (Table I). The total amount of haem present in secondary cultures of control cells also increased, but the increase was smaller and could be accounted for by an increase in cell number during secondary culture with a small proportion of spontaneous differentiation (Scher, Holland and Friend, 1971). The addition of BUdR to secondary cultures of cells previously exposed to DMSO resulted in a 50% decrease in the total amount of haem accumulated by the secondary cultures (Table I). The addition of BUdR to secondary cultures of control cells did not detectably inhibit spontaneous erythroid differentiation. This finding has been previously reported

TABLE I.—*Effect of BUdR on the Accumulation of Haem During Secondary Culture*

| | | Experiment | | |
|---|---|---|---|---|
| | | I | II | III |
| *Start of secondary culture | Control | 16 | 27 | 19 |
| | DMSO | 24 | 50 | 34 |
| †After 3 days secondary culture | Control | 56 | 209 | 212 |
| | Control + BUdR | 71 | 266 | 280 |
| | DMSO | 367 | 2030 | 1843 |
| | DMSO + BUdR | 180 | 1235 | 972 |

*$O.D._{415}$/10 ml of culture of Friend leukaemia cells at the start of secondary culture.

† $O.D._{415}$/10 ml of culture of Friend leukaemia cells at the end of secondary culture.

The $O.D._{415}$/10 ml of culture was calculated as follows: the $O.D._{415}/10^7$ cells was determined, and multiplied by: Cells/10 ml culture $\times 10^{-7}$

TABLE II.—*Effect of BUdR on Haem Synthesis After Exposure to and Removal from DMSO*

| | Exp. I | | Exp. II | | Exp. III |
|---|---|---|---|---|---|
| Cell count at 2d: | | | | | |
| Control | $6{\cdot}5\times10^5$ | | $11{\cdot}0\times10^5$ | | $8{\cdot}5\times10^5$ |
| DMSO | $2{\cdot}7\times10^5$ | | $8{\cdot}1\times10^5$ | | $3{\cdot}0\times10^5$ |
| $O.D._{415}/10^6$ cells at 2d: | | | | | |
| Control | $5{\cdot}22\times10^{-3}$ | | $8{\cdot}84\times10^{-3}$ | | $6{\cdot}23\times10^{-3}$ |
| DMSO | $8{\cdot}42\times10^{-3}$ | | $16{\cdot}7\times10^{-3}$ | | $11{\cdot}2\times10^{-3}$ |
| Cell count after secondary culture: | | | | | |
| Control | $1{\cdot}57\times10^6$ | | $3{\cdot}87\times10^6$ | | $3{\cdot}12\times10^6$ |
| Control+BUdR* | $2{\cdot}07\times10^6$ | | $4{\cdot}07\times10^6$ | | $3{\cdot}08\times10^6$ |
| DMSO† | $2{\cdot}7\times10^6$ | | $2{\cdot}82\times10^6$ | | $2{\cdot}88\times10^6$ |
| DMSO+BUdR‡ | $2{\cdot}7\times10^6$ | | $2{\cdot}52\times10^6$ | | $2{\cdot}7\times10^6$ |
| $O.D._{415}/10^6$ cells after secondary culture: | | % B+** cells | | % B+ cells | |
| Control | $3{\cdot}55\times10^{-3}$ | 0 | $5{\cdot}4\times10^{-3}$ | 0 | $6{\cdot}9\times10^{-3}$ |
| Control+BUdR | $3{\cdot}44\times10^{-3}$ | 0 | $6{\cdot}54\times10^{-3}$ | 1 | $9{\cdot}1\times10^{-3}$ |
| DMSO | $13{\cdot}6\times10^{-3}$ | 13 | $72{\cdot}0\times10^{-3}$ | 24 | $64{\cdot}0\times10^{-3}$ |
| DMSO+BUdR | $8{\cdot}2\times10^{-3}$ | 5 | $49{\cdot}0\times10^{-3}$ | 11 | $36{\cdot}0\times10^{-3}$ |

Note the difference in cell growth and haem synthesis between experiments I, II, and III. Different batches of foetal calf serum were used. Despite the variability in the absolute amount of haem synthesized, in all 3 experiments BUdR inhibited post-DMSO differentiation by approximately 50%.

* Control cells placed in secondary culture in the presence of BUdR.

† Cells which were exposed to DMSO during primary culture and placed in secondary culture in fresh DMSO-free medium.

‡ Same cells as above (†) but BUdR was present during secondary culture.

** Benzidine + ve.

using $^{59}Fe$ incorporation into haem as an index of haem synthesis (Scher, Preisler and Friend, 1973).

The addition of BUdR to secondary cultures of either control or DMSO-treated cells had no effect on the cell growth during the 3-day period of secondary culture (Table II). The amount of haem synthesized/cell in the culture and the proportion of benzidine-positive cells was decreased in each instance by approximately 50%. At first glance it seems surprising that the increment in the amount of haem/$10^6$ cells during secondary culture (*i.e.* the difference between the amount of haem present at the start and at the end of the secondary culture of cells exposed to DMSO during primary culture) appeared to be less than the increment in haem/culture. This observation arises because the rate of increase in differentiated cells during secondary culture was less than the rate of increase in undifferentiated cells in the same culture (Preisler and Giladi, 1975) and hence the proportion of differentiated cells in the secondary culture actually declined.

It has previously been demonstrated that BUdR interferes with the DMSO-induced differentiation of Friend leukaemia cells (Ostertag *et al.*, 1973; Scher *et al.*, 1973) and that this inhibition results from interference with the accumulation of globin mRNA (Conkie *et al.*, 1974; Preisler *et al.*, 1973). These observations, taken together with those reported here, strongly suggest that these leukaemic cells continue to synthesize and accumulate globin mRNA after removal of DMSO from the culture medium and that interference with this process inhibits differentiation during secondary culture in DMSO-free medium.

### REFERENCES

Conkie, D., Affara, N., Harrison, P. R., Paul, J. & Jones, K. (1974) *In situ* Localization of Globin Messenger RNA Formation. II. After Treatment of Friend Virus-transformed Mouse Cells with Dimethyl Sulfoxide. *J. Cell Biol.*, **63**, 414.

Friend, C., Scher, W., Holland, J. G. & Sato, T. (1971) Hemoglobin Syntheses in Murine Virus-induced Leukemic Cells *In vitro*. Stimulation of Erythroid Differentiation by Dimethyl Sulfoxide. *Proc. natn. Acad. Sci.* (*USA*), **68**, 378.

Ostertag, W., Crozier, T., Kluge, W., Melderis, H. & Dube, S. (1973) The Action of 5-Bromodeoxyuridine on the Induction of Hemoglobin Synthesis in Mouse Leukemia Cells Sensitive and Resistant to 5-BUdR. *Nature* (*New Biol.*), **243**, 203.

Preisler, H. D. & Giladi, M. (1975) Differentiation of Erythroleukemic Cells *In vitro*: Irreversible Induction by Dimethyl Sulfoxide (DMSO). *J cell. Physiol.*, **85**, 537.

Preisler, H. D., Housman, D., Scher, W. & Friend, C. (1973) Effects of 5-Bromo-2′-Deoxyuridine on Production of Globin Messenger RNA in Dimethylsulfoxide-stimulated Friend Leukemia Cells. *Proc. natn. Acad. Sci.* (*USA*), **70**, 2956.

Preisler, H. D., Scher, W. & Friend, C. (1973) Polyribosome Profiles and Polyribosome Associated RNA of Friend Leukemia Cells Following DMSO-induced Differentiation. *Differentiation*, **1**, 27.

Scher, W., Holland, J. G. & Friend, C. (1971) Hemoglobin Synthesis in Murine Virus-induced Leukemic Cells *In vitro*. I. Partial Purification and Identification of Hemoglobins. *Blood*, **37**, 428.

Scher, W., Preisler, H. D. & Friend, C. (1973) Hemoglobin Synthesis in Murine Virus-induced Leukemic Cells *In vitro*. IV. Effects of 5-Bromo-2′-Deoxyuridine, Dimethylformamide and Dimethylsulfoxide. *J. cell. Physiol.*, **81**, 63.